# Mid-Infrared Fiber-Coupled Photoacoustic Sensor for Biomedical Applications

**DOI:** 10.3390/s130100535

**Published:** 2013-01-02

**Authors:** Jonas Kottmann, Urs Grob, Julien M. Rey, Markus W. Sigrist

**Affiliations:** Institute for Quantum Electronics, ETH Zurich, Schafmattstrasse 16, 8093 Zurich, Switzerland; E-Mails: kjonas@phys.ethz.ch (J.K.); ursgrob@hotmail.com (U.G.); julien.rey@phys.ethz.ch (J.M.R.)

**Keywords:** photoacoustic, quantum cascade laser, spectroscopy, glucose

## Abstract

Biomedical devices employed in therapy, diagnostics and for self-monitoring often require a high degree of flexibility and compactness. Many near infrared (NIR) optical fiber-coupled systems meet these requirements and are employed on a daily basis. However, mid-infrared (MIR) fibers-based systems have not yet found their way to routine application in medicine. In this work we present the implementation of the first MIR fiber-coupled photoacoustic sensor for the investigation of condensed samples in the MIR fingerprint region. The light of an external-cavity quantum-cascade laser (1010–1095 cm^−1^) is delivered by a silver halide fiber, which is attached to the PA cell. The PA chamber is conically shaped to perfectly match the beam escaping the fiber and to minimize the cell volume. This results in a compact and handy sensor for investigations of biological samples and the monitoring of constituents both *in vitro* and *in vivo*. The performance of the fiber-coupled PA sensor is demonstrated by sensing glucose in aqueous solutions. These measurements yield a detection limit of 57 mg/dL (SNR = 1). Furthermore, the fiber-coupled sensor has been applied to record human skin spectra at different body sites to illustrate its flexibility.

## 1. Introduction

Optical fiber systems are widely used in the near infrared (NIR) where cheap silica based fibers offer a highly flexible and compact solution to deliver or collect light. Their application field is extremely broad reaching from medical imaging to the telecom industry. Due to the higher cost and lower quality (e.g., in terms of flexibility or transmission) mid-infrared (MIR) fibers have not yet found their way to routine applications in medical therapy and diagnostics. However, the demand for compact/portable, low-cost and reliable biomedical MIR fiber systems [1] or standoff detection schemes is immense [2]. For biomedical applications MIR fibers would be useful in therapy to deliver laser radiation (from quantum cascade lasers (QCL), CO_2_-, CO- or Er:YAG lasers) or in diagnostics to record a thermal image of the body or a tissue spectrum (e.g., for optical biopsy or monitoring of tissue constituents) in the MIR fingerprint region [3–5]. Especially, QCLs have boosted this request since they offer room temperature operation, a narrow linewidth, reasonable average power, a compact design and emission in the 3–20 *μ*m wavelength range.

So far, there have been only few attempts of directly coupling a fiber to a photoacoustic (PA) cell. These are all related to trace gas detection and employ mostly fiber-coupled laser diodes [6,7]. Schilt *et al.* for example employed a distributed feedback (DFB) laser diode emitting at 1.65 *μ*m to detect methane by coupling the light via a fiber directly into a resonant PA cell [8]. A MIR-fiber was used by Elia *et al.* to couple a pulsed QCL into a PA cell directly attached to the fiber to measure formaldehyde (CH_2_O) at 1,778.9 cm^−1^ [9]. However, to the best knowledge of the authors, the present work is the first one where a fiber-coupled PA cell was employed to investigate liquids and solids in the MIR. Despite current limitations of MIR fibers, we implemented a MIR fiber-coupled PA sensor and demonstrated its capability to detect solutes like glucose in biological samples. The sensor combines the advantages of a sensitive detection based on PA spectroscopy with the flexibility offered by a fiber-coupled system, which is especially advantageous for *in vivo* applications. The rigid fixation of the fiber to the PA cell ensures a fixed location of the light excitation even if the PA cell is moved. In addition our PA sensor is advantageous because of its small PA chamber volume of only 35 mm^3^. This is enabled by the fiber coupling and the conical shape of the chamber. A further specialty of the PA cell design is the integrated N_2_ ventilation system, which provides constant conditions in the PA chamber, even if samples containing volatile compounds like water (typically present in biological samples) are investigated [10,11]. The performance of the fiber-coupled PA sensor is demonstrated by the detection of glucose in aqueous solution. These measurements led to a detection limit of 140 mg/dL for an integration time of 1 s (57 mg/dL for an integration time of 45 s) and a signal-to-noise ratio (SNR) of one. This lies within the physiological range (*i.e.*, 30–500 mg/dL), but needs to be improved if non-invasive *in vivo* glucose measurements should become feasible in the future. Such a non-invasive PA sensor would facilitate the life of diabetes patients considerably if it were able to measure glucose reliably with an accuracy of ±15 mg/dL. By exploiting the tuning capability of the QCL and the flexibility of the fiber-coupled PA cell, *in vivo* spectra of human skin were recorded at the finger tip and the forearm.

## 2. MIR Fibers

Unfortunately, glass fibers based on silica and fluoride are only transparent up to 6 and 7 *μ*m, respectively [12]. Some chalcogenide glasses transmit light up to 15 *μ*m, but they exhibit a low transmission for longer wavelengths and rather poor mechanical properties. Furthermore, some of them are even toxic or water soluble and hence do not represent a valid option [3]. MIR fibers have also been produced out of two different crystal groups: thallium halides (TlClBr) and silver halides (AgClBr). Since thallium halides are extremely toxic and rather unstable, silver halide crystals are usually the material of choice for the production of MIR-fibers [12].

Single silver halide crystals have a composition of AgCl*_x_*Br_1−_*_x_* (0 ≤ x ≤ 1) and are highly transparent in the 3 to 30 *μ*m wavelength range, what makes them useful for a broad range of applications. The refractive index almost linearly decreases with increasing x from 2.16 for AgBr to 1.98 for AgCl. This enables the fabrication of core-clad fibers [13]. The crystals’ mechanical and optical properties (hardness, melting point, light sensitivity, *etc.*) vary according to its composition. The fibers are typically produced out of a single crystalline rod by extrusion through a small die. The resulting polycrystalline fibers (grain size approximately 1 *μ*m [12]) are reasonably flexible, nontoxic, biocompatible and very slightly soluble in water. Furthermore they exhibit an acceptable low sensitivity to ultraviolet and visible light and have typical transmission losses of 0.2 dB/m at 10.6 *μ*m [14].

Two different types of fibers exist: core-only and core-cladding fibers. These are typically fabricated as multimode fibers with core diameters between 300 and 800 *μ*m. Recent progress has been made towards step-index few-modes [13] and even single mode fibers [15–17]. In order to obtain single mode operation in a silver halide fiber, a core radius of between 15 and 35 *μ*m is required, which is challenging to fabricate [17] and exhibits high transmission losses of 15 to 20 dB/m (at 10.6 *μ*m). The concept of another fiber class, called photonic crystal fibers, can be employed to construct single-mode silver halide waveguides. These fibers possess lower losses and provide a larger mode diameter (∼100 *μ*m) [16].

Silver halide fibers of different design (*i.e.*, cylindrical, tapered, flattened or U-shaped) have been widely employed in evanescent wave spectroscopy [12]. For such investigations a part of the fiber core is exposed to the sample. In this region the light, coupled into the fiber, interacts via the evanescent field with the sample. Particularly in the analysis of biological samples like blood, tissue, glucose, albumin or urine, such non-destructive Attenuated Total Reflection (ATR) techniques have been used [1,18]. However, in PA spectroscopy the fiber is solely required to deliver the laser light to the PA cell.

Another MIR fiber class is hollow core optical fibers, which are less fragile and do not exhibit cladding modes compared with the solid-core fibers. The fibers are fabricated out of a hollow glass capillary tube. With wet chemistry techniques the glass tubes are coated on the inner surface with a dielectric silver iodide (AgI) layer on top of a reflective silver (Ag) layer [19]. The light is then guided in a hollow core with a diameter of typically 300–800 *μ*m through reflection. Despite the large core diameter single mode transmission is possible in these fibers as demonstrated by Kriesel *et al.* [19]. Recently the transmission of hollow core fibers for QCL radiation in the 9 to 10 *μ*m wavelength range have been investigated and losses of down to 0.44 dB/m have been reported [20,21]. Employing a hollow-core fiber-coupled QCL sensitive spectroscopic measurements of SF_6_ (normalized noise-equivalent absorption of 2.7 × 10^−10^ W·cm^−1^/Hz^1/2^) have been conducted by Spagnolo *et al.* using a quartz enhanced photoacoustic spectroscopy (QEPAS) technique [22].

## 3. Photoacoustic Spectroscopy

**Figure 1.**
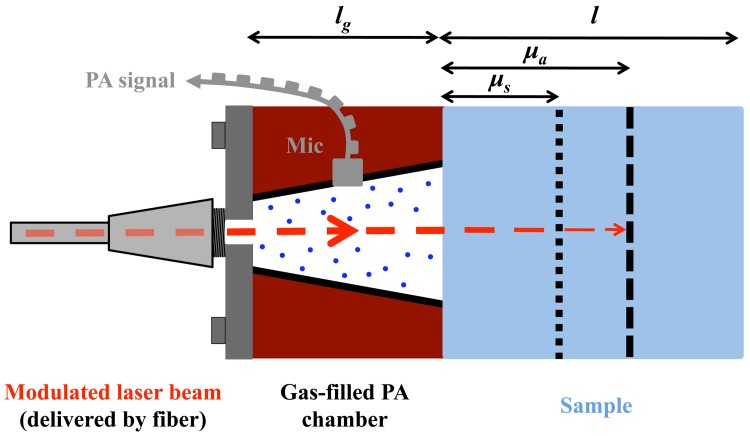
Illustration of the PA signal generation. According to the Rosencwaig–Gersho model [23] the length ratios between the sample length *l*, the optical absorption length *μ_a_ =* 1/*α* and the thermal diffusion length *μ_s_* are used to distinguish different cases. In this figure a length ratio of *l* > *μ_a_* > *μ_s_* is pictured, as in the samples studied in this work. The periodical PA signal is detected with a microphone (Mic) in the gas-filled PA chamber of length *l_g_*.

Photoacoustic spectroscopy is a well established sensitive technique used in investigations of gases, liquids and solids. It is applied in a broad field ranging from physics, chemistry, biology and material sciences to medicine. In PA spectroscopy the sample is irradiated with modulated light usually from a laser. The absorbed optical energy is converted to heat by means of non-radiative relaxation of the molecules and creates an acoustic wave in the sample. In a solid, the acoustic wave can either be sensed by a piezoelectric sensor at the surface or by a microphone in the coupling gas of an adjacent PA cell as in this work (see Figure 1).

For the study of solid samples with a PA cell, Rosencwaig and Gersho developed a model according to which 6 different cases of PA signal generation can be distinguished depending on the ratio between the sample length *l*, the optical absorption length *μ_a_=* 1/*α* (*α*: absorption coefficient) and the thermal diffusion length of the sample *μ_s_* (not to be confused with the symbol for the scattering coefficient) [23]. All these cases have an identical dependence of the PA signal amplitude on the properties of the coupling gas and the light intensity. Using the same notation as Tam [24] and in addition the wavelength dependent fiber transmission *t*(*λ*) we summarize this dependence using a factor (*F*) defined as:

(1) $$F=\frac{\gamma \cdot P_{0}\cdot t(\lambda )\cdot I_{0}\cdot \mu_{g}}{4\sqrt{2}\cdot l_{g}\cdot T_{0}}$$

where *γ* = *C_p_*/*C_v_* is the ratio of the specific heat at constant pressure and volume, *P*_0_ the ambient pressure, *I*_0_ the amplitude of the laser intensity, *μ_g_* the thermal diffusion length of the coupling gas, *l_g_* the length of the coupling gas and *T*_0_ the ambient temperature. The thermal diffusion length of the sample (*s*) and coupling gas (*g*) are defined as:

(2) $$\mu_{g,s}={\left(\frac{D_{g,s}}{\pi \cdot f}\right)}^{\frac{1}{2}}$$

where *f* is the modulation frequency of the laser and $D_{i}=\frac{k_{i}}{\rho_{i}C_{p?}}$ the thermal diffusivity, with *k_i_* being the thermal conductivity and *ρ_i_* the density of the coupling gas (*g*) or sample (*s*), *i.e.*, *i* = *g* or *s*.

For a biological sample like human skin or aqueous solutions as considered here, which are both characterized by high water content, the penetration of MIR light is small due to the strong water absorption in this wavelength region. Hence, the sample length *l* is usually larger than the optical penetration depth *μ_a_* (*i.e.*, *l* > *μ_a_*) as indicated in Figure 1. By adjusting the modulation frequency *f* one can vary the thermal diffusion length of the sample *μ***_s_** (see Equation (2)) and restrict the discussion of the Rosencwaig–Gersho model to the case in which *l* > *μ_a_* > *μ_s_* holds. In this case the PA signal amplitude (A*_PA_*) dependence is given by:

(3) $$A_{PA}\propto \frac{t(\lambda )\cdot I_{0}\cdot \alpha }{V\cdot f^{\frac{3}{2}}}$$

where *V* denotes the PA cell volume and where the dependence on parameters summarized in *F* (see Equation (1)) are included.

## 4. Experimental Section

**Figure 2.**
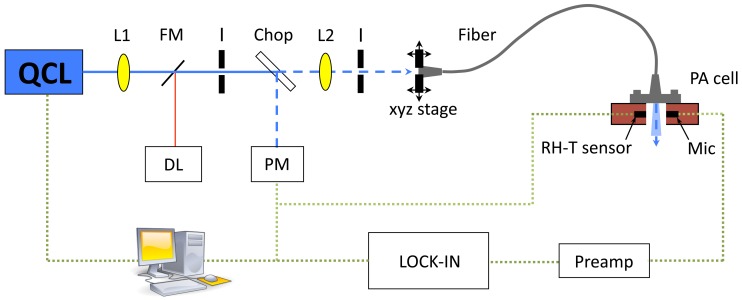
Setup for photoacoustic measurements: QCL = quantum cascade laser, FM = flipping mirror, L = lens, I = Iris, DL = diode laser (for alignment tasks), Chop = chopper, PM = power meter, Mic= microphone and RH-T = relative humidity-temperature sensor.

A sketch of the PA setup is displayed in Figure 2. The emission of the external-cavity quantum cascade laser (EC-QCL) (Daylight Solutions DLS-TLS-001-PL) covers the wavelength range from 1,010–1,095 cm^−1^. This includes the two strong glucose absorption peaks at 1,034 and 1,080 cm^−1^. The EC-QCL is tunable via the external grating in steps of 0.9 cm^−1^ and a fine tuning can be obtained by changing the QCLs chip temperature or current. The maximal average laser power depends on the emission wavelength and ranges from 13 mW at 1,010 cm^−1^ to 125 mW at 1,055 cm^−1^. The continuous-wave (cw) laser beam is modulated by a mechanical chopper (New Focus Model 3501) with gold-coated blade at ∼125 Hz and focused by two anti-reflection coated ZnSe lenses into the silver halide fiber mounted on a xyz-stage. To deliver the QCL light to the PA cell, two different step-index silver halide fibers (CeramOptec GmbH) with a core diameter of 400 and 600 *μ*m, respectively, have been employed. Both fibers are 0.7 m ± 0.05 long, have a numerical aperture of 0.13 ± 0.02 and a cladding diameter, which exceeds the core diameter by 100 *μ*m. The minimum bend radius *R* equals 100 times the fiber diameter. The multimode fibers have Ti-SMA-connectors at both ends to ease the connection to the sensing head and are protected with a polyether ether keton (PEEK) tube, which impedes a too strong bending. The core refractive index is 2.1 and the transmission range of the fiber reaches from 4 to 16 *μ*m. The beam emerging the fiber then passes the PA chamber and is absorbed in the sample sealing the cell. The generated acoustic wave is sensed with an electret microphone (Knowles FG-23329-P07) in the coupling gas (air and N_2_ mixture). The small microphone diameter (2.59 mm) allows a compact cell volume *V* which is favorable in terms of signal amplitude (see Equation (3)) and sensor size. The pre-amplified PA signal is measured with a lock-in amplifier (Stanford Research SR830) before being recorded with a computer. To monitor the conditions in the PA chamber a compact temperature (T) and relative humidity (RH) sensor (Sensirion SHT21, 3 mm × 3 mm × 1.1 mm) was integrated.

### 4.1. Photoacoustic Cell Design

**Figure 3.**
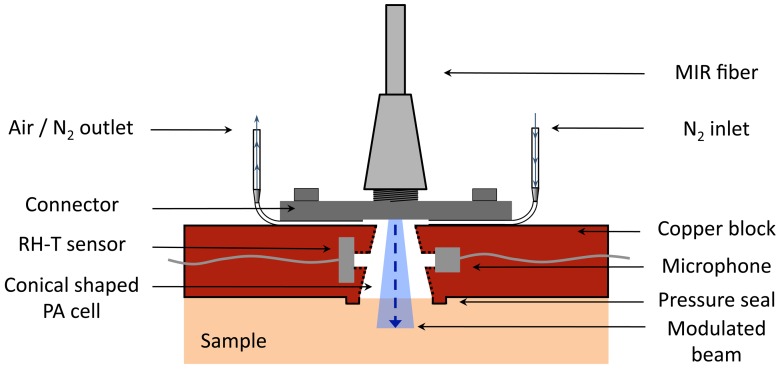
Schematic of the fiber-coupled PA cell. The PA chamber is conically shaped and closed directly by the sample itself. N_2_ ventilation is required, if a sample containing volatile components like water is investigated.

The design of the fiber-coupled PA cell for the investigation of biological samples is pictured in Figure 3. The PA cell is manufactured out of a copper piece of 4 × 25 × 35 mm. A conically shaped hole (upper diameter 2.1 mm and lower diameter 4.0 mm) builds the PA chamber, which ideally encloses the divergent QCL beam escaping the fiber. This design enabled to reduce the PA chamber volume *V* to 35 mm^3^. In comparison with PA cells described in former publications [10,11,25], this represents a volume reduction by a factor > 2. Since the PA amplitude for a non-resonant cell is proportional to *V*^−1^ (see Figure 3), a small volume is preferred. The inner surface of the PA cell has been polished and gold coated to minimize the undesired PA signal from the cell itself. On the upper end the PA chamber is directly closed by a home-built aluminum SMA fiber connector, which is screwed onto the PA cell. At the bottom side of the connector two little grooves have been incorporated, into which two thin needles (length =19 mm and inner diameter = 0.4 mm) were placed and fixed with glue to ventilate the PA chamber with N_2_. One needle is connected via silicon tubes to a mass flow controller (MKS Instruments, Multi Gas Controller 647B) providing a constant flow of N_2_ to the cell while the other is left open. This ensures stable conditions in the PA chamber essential for precise measurements if samples containing volatile components are investigated (for details see references [10,11]). The MIR fiber is simply screwed to the connector and delivers the modulated light to the sample sealing the PA chamber on the lower side. The rigid fixation of the fiber to the PA cell guarantees a stable location of the excitation beam with respect to the cell even if it is moved. To improve the sealing of the PA cell by the sample a small ring structure of 0.5 mm height (called “pressure seal” in Figure 3) was integrated in the cell design. The generated PA signal is then sensed with a microphone, which is connected through a cylindrical hole (length = 1 mm, diameter = 1 mm) with the main PA chamber. Similarly, an RH-T sensor is integrated on the opposite to monitor the conditions in the PA chamber. Optionally the PA cell can be closed on the lower end with a chemical vapor deposition (CVD) diamond membrane (3.91 *μ*m thick, Diamond Materials) to enable the study of liquids. Due to the outstanding thermal and optical properties of diamond, a strong PA signal of the liquid of interest brought in direct contact with the diamond membrane is obtained [25].

## 5. Results and Discussion

### 5.1. Transmission of the MIR Fiber

**Figure 4.**
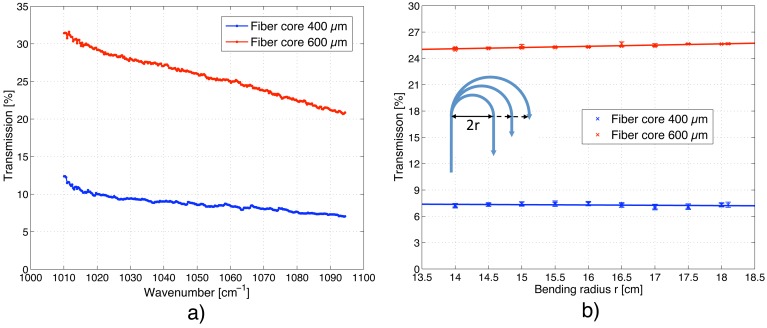
(**a**) Wavelength dependence of the transmission of the MIR fiber; (**b**) Variation of the fiber transmission at 1055 cm^−1^ upon changing the bending radius of the fiber.

The transmission of silver halide fibers covers a broad wavelength range (3–30 *μ*m) but it is not entirely flat due to impurity bands near 2.9, 6.28 and 7.15 *μ*m and scattering losses [26]. Unlike in silica fibers, where the scattering losses vary with 1/*λ*^4^ (Rayleigh scattering), the scattering in silver halide fibers decreases for longer wavelength with 1/*λ*^2^ [27]. In order to determine the laser intensity exciting the sample, the transmission *t*(*λ*) of both MIR fibers was measured. With a coarse fiber coupling, a transmission of 32–21% could be achieved for the 600 *μ*m core fiber, whereas only 13–7% could be transmitted through the 400 *μ*m core fiber (see Figure 4(a)). Both fibers show a similar wavelength dependence of the transmitted intensity, which is higher for longer wavelengths and decreases almost linearly towards shorter wavelengths. These transmittance curves are required to power-normalize the PA signal according to Equation (3). This is particularly important when recording a spectrum, since the output power of the laser varies substantially over the tuning range (*i.e.*, 10–125 mW).

The transmission of the MIR fiber in the elastic bending regime (*i.e.*, bending radius >100 times the core diameter) was investigated by curving the fiber in a half circle as displayed in the inset of Figure 4 (b). The transmission was measured at 1,055 cm^−1^ and the radius was varied between 14 and 18 cm in 0.5 cm steps. Within this bending range the transmission for both fibers remains constant (see Figure 4 (b)). The ratio of the standard deviation to the mean of the transmission for measurements at different fiber bending is less than 0.9% for the 600 *μ*m and 1.9% for the 400 *μ*m core fiber. However, the variation is ≤ 0.4% if measurements are performed at a fixed fiber bending.

### 5.2. Glucose Detection in Aqueous Solutions

The open-ended PA cell is well suited to study solid samples, which provide a rigid closure of the PA chamber. However, the investigation of liquid samples (e.g., glucose solution) with such a design is difficult since small movements of the liquid might alter both the PA chamber volume and the sample surface, which results in unstable PA signals. Hence, to study liquid samples the cell needs to be closed with a thin diamond membrane or window as described in previous works [10,25]. Closing the PA cell with a diamond optic ensures a stable and strong signal due to the high optical transparency and the good thermal conductivity of diamond. The liquid sample is placed outside the PA chamber in good thermal contact with the diamond membrane closing the PA cell.

**Figure 5.**
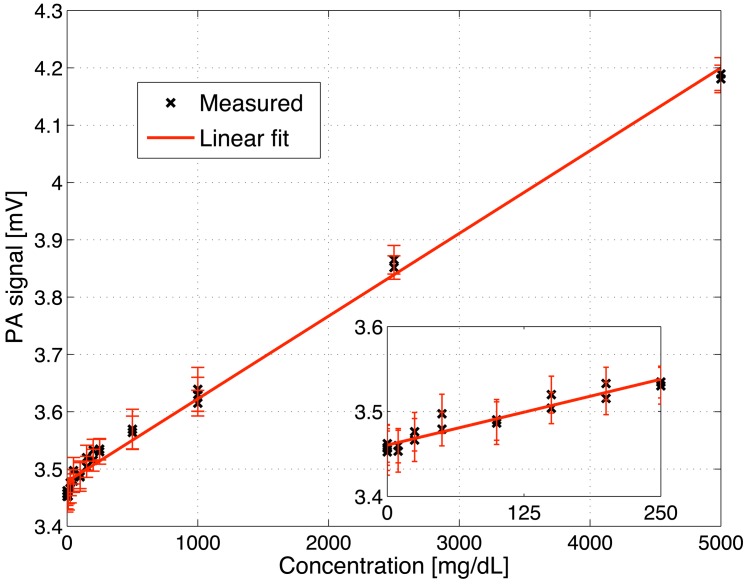
PA signal versus glucose concentration in aqueous solutions. The measurements were conducted a 1,034 cm^−1^ with an integration time of 1 s and a modulation frequency of 117 Hz. The error bars correspond to twice the standard deviation (*i.e.*, ± *σ*) using a lock-in integration time of 1 s. The inset depicts the concentration range 0–250 mg/dL.

To determine the glucose detection limit of the fiber-coupled PA cell, different glucose solutions with concentrations ranging from 0 to 5 g/dL were prepared and consecutively placed on the diamond membrane. The measurements were performed at the glucose absorption peak at 1034 cm^−1^ with a modulation frequency of 117 Hz. The recorded PA signal linearly increases with glucose concentration within the entire studied range (R^2^ = 0.993) as seen in Figure 5. In the inset a magnification of the low concentration range (0–250 mg/dL) is shown. The error bars correspond to twice the standard deviation (*i.e.*, ± *σ*) using a lock-in integration time of 1 s. The obtained detection limit is 140 mg/dL for a SNR = 1. This lies a factor 4.2 higher than the detection limit achieved with a non-fiber-coupled PA sensor reported earlier in [25]. However, averaging the PA signal over a longer period of 45 s results in a root mean square error of only 8.6 *μ*V, which yields a detection limit of 57 mg/dL for a SNR = 1. The currently worse detection limit of the fiber-coupled PA sensor is mainly caused by two reasons: the reduced excitation power (between 2 and 15 mW) and the light transmission instability caused by the MIR fiber (compare Figure 4). For *in vivo* application the low transmitted power does not pose a problem since the maximal permitted cw irradiation of human skin with MIR light is anyway limited to 1 mW/mm^2^ for MIR light between 2.6 and 10 *μ*m [28]. However, for in vitro studies omitting the fiber and directly coupling the light to the PA cell can increase the detection sensitivity due to the higher available excitation power.

### 5.3. Spectra of Glucose in Aqueous Solutions

**Figure 6.**
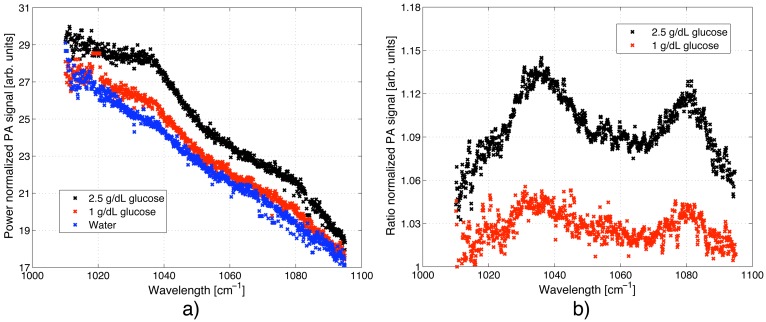
PA spectrum of an aqueous solution with 0, 1.0 and 2.5 g/dL glucose. The QCL was tuned via the external grating and modulation with a frequency of 117 Hz. (**a**) The PA signal was normalized with the output power of the QCL. The wavelength dependent transmission of the fiber was not taken into account, which results in a reduced PA signal for larger wavenumbers; (**b**) Taking the ratio between the glucose and water spectrum reveals the two glucose absorption peaks at 1,034 and 1,080 cm^−1^.

Exploiting the tuning capability of the EC-QCL enables to record sample spectra. For liquids and solids a coarse tuning via the external grating (resolution ∼0.9 cm^−1^) is often sufficient to resolve the broad spectral signatures. In Figure 6(a) the PA spectrum of a 0, 1.0 and 2.5 g/dL glucose solution is displayed normalized with respect to the output power of the laser. For these measurements the QCL was modulated with 117 Hz and the grating was tuned in small steps with an average of 9 grating positions per emission wavelength (e.g., 9 grating steps before the laser emission jumps to the next cavity mode). All spectra show a decreasing signal for larger wavelengths since the wavelength dependent transmission of the fiber has not been taken into account. The increased noise close to 1,010 cm^−1^ is caused by the low laser intensity (∼2 mW) exciting the sample in this wavelength range.

Taking the ratio between the glucose spectrum and the water spectrum is a convenient way to eliminate the wavelength dependence of the fiber transmission *t*(*λ*) (compare Equation (3)). The resulting glucose spectrum with its two absorption peaks centered at 1,034 and 1,080 cm^−1^ is clearly visible in Figure 6(b).

### 5.4. In vivo Spectra of Human Skin

**Figure 7.**
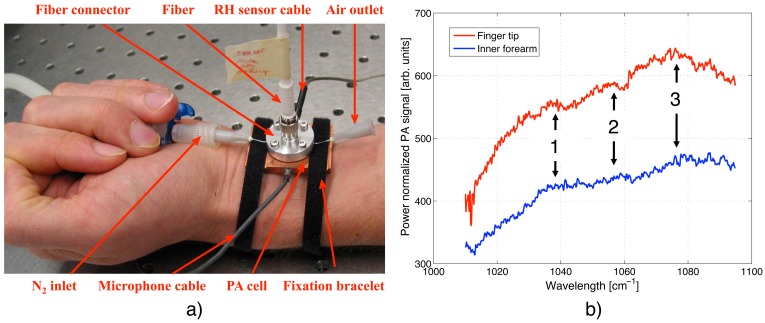
(**a**) Picture of the fiber-coupled PA sensor attached with hook-and-loop stripes to the forearm; (**b**) PA spectrum of human skin measured at the finger tip and the forearm. Possible contributions to the absorption peaks numbered 1–3 are listed in Table 1.

To fully exploit the flexibility offered by the fiber-coupled PA sensor, human skin spectra at the finger tip and the forearm have been recorded. For these studies, either the sensor head was fixed with hook-and-loop stripes at the body site as seen in Figure 7(a) for the forearm, or alternatively, the body part was pressed on the PA sensor opening to seal the chamber at the lower end. Due to the strong water absorption in the MIR, the penetration depth of the QCL light into human skin is limited to < 100 *μ*m. Hence, only the horny surface layer (stratum corneum) and the underlying not by blood perfused epidermis can be reached with MIR light and contribute to the spectral signature shown in Figure 7(b). Even though not perfused by blood, variations in the glucose level can still be tracked by accessing only the epidermal skin layer since glucose diffuses out of the blood vessels into the interstitial fluid of the epidermis. As before, the QCL was tuned step-wise via the external grating and modulated with 137 Hz. Generally, the PA signal generated at the finger tip was considerably stronger than that measured at the forearm. This can be explained by the different structure of the skin at these two measurement sites. At the finger tip, the thickness of the stratum corneum – exhibiting a water content of only 10% – can exceed 1 mm, whereas it is usually just 10 to 20 *μ*m at the forearm. This results in a deeper light penetration into the skin and a smaller signal contribution from the underlying epidermis (water content ∼60%) at the finger tip. However, the spectral signature at both measurement sites is similar with three broad absorption peaks centered at ∼1,037, 1,055 and 1,078 cm^−1^. Due to the complexity of human skin, an assignment of the individual absorption peaks observed is difficult since nucleic acids, carbohydrates, lipids and proteins exhibit characteristic vibrations in the studied wavelength range [29]. Amongst others, molecular vibrations of *α*— and *β*— D-glucose leading to absorption peaks at 1,034, 1,054 and 1,080 cm^−1^ [25] and of albumin resulting in absorption signatures at 1,020 and 1,052 cm^−1^ [30] might contribute to the spectrum of skin within the investigated region. According to Garidel [31] and Lucassen *et al.* [32] the absorption peak at 1,078 cm^−1^ can originate from a *ν*(CC) skeletal *trans* conformation (1,077 cm^−1^) and/or a ${\text{PO}}_{2}^{-}$ symmetric stretch. The exact position of the ${\text{PO}}_{2}^{-}$ stretch is strongly influenced by the presence of cations and hydration effects, which cause variations in its position of ± 4 cm^−1^. Further contributions to the overall spectral structure arise from the *ν*(CC) *cis* conformation at 1,035 cm^−1^ and the C–OP stretch at 1,047 cm^−1^ [31]. All these contributions found in literature are summarized in Table 1 together with an indication of their possible influence on the skin spectra shown in Figure 7 (b). The strong characteristic glucose absorption at 1,034 and 1,080 cm^−1^ observed in aqueous solutions (see Figure 6) could contribute to the spectral signature of peak 1 and 3 in Figure 7(b), whereas its influence on peak 2 at ∼1,055 cm^−1^ is small (compare Table 1). To confirm a spectral contribution of glucose, spectra of different glucose concentrations would need to be recorded. However, a comparison of the *in vivo* peak positions with those found in earlier *in vitro* studies on epidermal skin [11] show similarities and demonstrate the capability of the fiber-coupled PA sensor.

## 6. Conclusions

In this work, the first implementation of a fiber-coupled PA sensor for the investigation of solid and liquid samples was presented. By employing a MIR silver halide fiber and an EC-QCL, first measurements were performed in the interesting MIR fingerprint region. The combination of the PA technique and the fiber-coupling results in a sensitive, flexible and compact sensing device. This simplifies the use of the PA sensor at various measurement locations, which is particularly interesting for medical applications. Currently the MIR fiber coupling still leads to a loss in sensitivity compared with a conventional free-space light coupling mainly for two reasons: the low transmission and small fluctuations in the transmitted intensity of the silver halide fibers. Nevertheless, with today's rapid progress in MIR fiber fabrication technology, these limitations should be considerably reduced within the years to come. However, the implementation of a fiber-coupled PA cell is not limited to the MIR wavelength region as employed in these studies. Already today, by exploiting established NIR optical fibers, which are cheaper, more flexible and exhibit higher and more stable transmission, no significant loss in sensitivity should be caused by a fiber-coupling.

To demonstrate the capability of the fiber-coupled PA sensor, glucose was monitored in aqueous solutions within the concentration range from 0 to 5 g/dL. The obtained detection limit was 140 mg/dL using an integration time of 1 s and 57 mg/dL with an integration time of 45 s for a SNR= 1. This is currently higher as compared with an identical PA cell without fiber coupling and needs to be further improved to enable an *in vivo* application of the sensor. However, with an improved detection limit, a fiber-coupled PA sensor appears feasible for non-invasive glucose monitoring in the future. This would clearly ease the life of millions of diabetes patients.

*In vivo* investigations of the flexible fiber-coupled sensor have been demonstrated by measurements at different body sites. However, the current sensitivity is still too low for actual *in vivo* glucose detection and further studies to evaluate potential interfering effects in human skin would be needed. Nevertheless, our studies represent an important contribution to meet the requirements of non-invasive glucose sensing for diabetes patients.

## Figures and Tables

**Table 1.** Vibrational absorption bands of skin found within the tuning range of the QCL according to literature [30–33]. Used abbreviations: v = very, m = medium, w = weak and *ν* = stretch. The numbering 1 to 3 indicates their possible contribution to the absorption peaks observed in the skin spectra displayed in Figure 7(b).

**Frequency [cm**^−1^]	**Assignment**	**Strength**	**Contribution**
1020	albumin absorption	vw	-
1034	*α* & *β* D-glucose absorption	m	1
1035	*ν*(CC) skeletal *cis* conformation	m	1
1047	*ν*(C-OP)	w	2
1052	albumin absorption	w	2
1054	*α* D-glucose absorption	vw	2
1077	*ν*(CC) skeletal *trans* conformation	m	3
1080	${\nu (\text{PO}}_{2}^{-})$ symmetric	m	3
1080	*β* D-glucose absorption	m	3
